# Sources of intraspecific variation in the isotopic niche of a semi-aquatic predator in a human-modified landscape

**DOI:** 10.7717/peerj.15915

**Published:** 2023-08-30

**Authors:** André Costa Pereira, Gabriela Bielefeld Nardoto, Guarino Rinaldi Colli

**Affiliations:** 1 Departamento de Zoologia, Instituto de Ciências Biológicas, Universidade de Brasília, Brasilia, Distrito Federal, Brazil; 2 Departamento de Ecologia, Instituto de Ciências Biológicas, Universidade de Brasília, Brasilia, Distrito Federal, Brazil

**Keywords:** Anthropogenic habitats, *Caiman crocodilus*, Ontogenetic shifts, Sexual niche variation, Niche temporal variability

## Abstract

Intraspecific variation modulates patterns of resource use by species, potentially affecting the structure and stability of food webs. In human-modified landscapes, habitat disturbance modifies trophic interactions and intraspecific niche variation, impacting population persistence. Here, we investigated the relationship of sex, ontogeny, and habitat factors with the trophic niche of *Caiman crocodilus* in an agricultural landscape. We evaluated temporal variation in the trophic niche parameters using carbon and nitrogen stable isotope analysis from different body tissues. We found that caimans exploit the same carbon and nitrogen pools through time, with low isotopic variability between seasons, partly due to the slow isotope turnover rates of tissues in crocodilians. Conversely, the trophic niche of caimans varied across habitats, but with no evidence of a difference between natural and anthropogenic habitats. It apparently results from the influence of habitat suitability, connectivity, and caiman movements during the foraging. Our findings highlight the broader niches of juvenile caimans relative to adults, possibly in response of territorialism and opportunistic foraging strategy. Although using similar resources, females had a larger niche than males, probably associated with foraging strategies during nesting. Considering the sex and body size categories, caimans occupied distinct isotopic regions in some habitats, indicating apparent niche segregation. Ontogenetic trophic shifts in the isotopes (*δ*^13^C and *δ*^15^N) depended on sex, leading to resource partitioning that can potentially reduce intraspecific competition. Decision-makers and stakeholders should consider the trophic dynamics of sex and body size groups for the sustainable management and conservation of caiman populations, which implies in the maintenance of wetland habitats and landscape heterogeneity in the Formoso River floodplain.

## Introduction

Intraspecific variation has significant ecological effects on populations, communities, and ecosystems, mainly acting as the raw ingredient of natural selection (when heritable) and a factor that promotes coexistence (Bolnick et al., 2011; Violle et al., 2012). Despite receiving diminished attention at the turn of the century, interest in intraspecific variation has resurged. It is considered as essential as the variation among species in community assembly and dynamics (Des Roches et al., 2018). Furthermore, intraspecific trait variation influences population dynamics, community assembly, and ecosystem functioning through ecological and evolutionary processes. For instance, intraspecific trait variation affects predator-prey interactions at the individual and population levels, which in turn can modify the structure and stability of food webs (Araújo, Bolnick & Layman, 2011; Bolnick et al., 2003; McCann, Rasmussen & Umbanhowar, 2005). Through phenotypic plasticity and contemporary evolution, human activity is a powerful driver of change in species traits, implying dramatic and harmful ecological effects (Fryxell & Palkovacs, 2017; Palkovacs et al., 2012). Therefore, understanding intraspecific variation is crucial to predicting population processes and dynamics in the face of changing environments and promoting successful management and conservation of species.

Intraspecific variation is evident in species’ sexual and body size/age traits (Polis, 1984; Shine, 1989). By reducing intraspecific competition, such variation can drive habitat segregation and coexistence (Wearmouth & Sims, 2008; Woodward et al., 2005). Body size is a structural-functional regulator of food webs, trophic interactions, and other ecological processes (Rooney, McCann & Moore, 2008; Woodward et al., 2005). Ontogenetic shifts in metabolic rates and energetic requirements with increasing body size can generate functionally separate groups with distinct ecological niches (Dunic & Baum, 2017). Hence, niche width and trophic level can positively relate to body size due to a predator-prey size relationship in size-structured food webs (Arim et al., 2010). In the same way, sexual differences in predation risk, dimorphism, physiological requirements, forage selection, and activity budgets can drive differences in resource and habitat use, causing sexual segregation (Beck et al., 2007; Lima et al., 2019; Nifong, Layman & Silliman, 2015; Smith et al., 2015; Wearmouth & Sims, 2008).

Trophic ecology has gained recent impetus through stable isotope analysis (SIA) developments. The quantification of carbon (*δ*^13^C) and nitrogen (*δ*^15^N) stable isotopes, incorporated through the diet in body tissues across the lifespan of an organism, enables insights into diet composition and trophic relationships through the calculation of an isotopic niche in multivariate *δ*-space (Newsome et al., 2007). *δ*^13^C and *δ*^15^N vary spatially and temporally in food webs, enabling inferences on the basal energy source *via δ*^13^C and the trophic level *via δ*^15^N (Boecklen et al., 2011). Thus, SIA can inform various aspects of an animal’s trophic ecology, including dietary and habitat use, specialization, movement patterns, trophic coupling, and anthropogenic impact on the food web (Layman et al., 2007b; Newsome et al., 2012; Rosenblatt & Heithaus, 2011). For these applications, SIA enables characterizing and quantifying trophic niche properties, such as evenness and packing, width, position or trophic length, resource diversity, degree of overlap, dietary variation, and niche path trajectories (Bearhop et al., 2004; Hammerschlag-Peyer et al., 2011; Jackson et al., 2011; Layman et al., 2007a; Newsome et al., 2012; Turner, Collyer & Krabbenhoft, 2010). Moreover, tissues with different turnover rates from the same individuals allow resource use analyses at multiple temporal and spatial scales (Dalerum & Angerbjörn, 2005; Martínez del Rio et al., 2009). For instance, metabolically active tissues (*e.g*., plasma, liver) convey short-term dietary information, while inert tissues (*e.g*., scute, claw, hair) incorporate and reflect a long-term consumption related to their specific formation time (Vander Zanden et al., 2015). Therefore, SIA can help understand the intraspecific variation associated with body size and sex traits.

Isotopic parameters can reveal spatiotemporal patterns of niche variation (Hammerschlag-Peyer et al., 2011; Marques et al., 2013; Turner, Collyer & Krabbenhoft, 2010). For example, the seasonal flood pulse of wetlands changes the isotopic ratio values of basal sources, promoting isotopic niche shifts among consumers (Sepúlveda-Lozada et al., 2017; Wantzen, Fellerhoff & Voss, 2010). Anthropogenic disturbances can alter resource availability and foraging behavior, causing either niche expansion and high niche overlap (Manlick & Pauli, 2020; Swihart et al., 2006; Zambrano, Valiente & Vander Zanden, 2010) or a reduction of niche width and trophic level (Burdon, McIntosh & Harding, 2020; Layman et al., 2007b). In addition, the potential increase in competition causes the extirpation of sensitive species, affecting community structure and the food web (Le Provost et al., 2020). Further, human-induced trait changes can alter processes (migration, movement, maturation, and habitat selection) that reflect individual fitness and population persistence and trigger non-natural eco-evolutionary dynamics, including phenotypic plasticity and contemporary evolution (Palkovacs et al., 2012). The magnitude of the anthropogenic impacts on the food web can become even more severe when they fall on a top predator species through trophic cascades (Estes et al., 2011).

Crocodilians are top predators and play a crucial role in maintaining the structure and functioning of food webs in diverse ecosystems. Their intraspecific trait variation regulates the strength and dynamics of trophic relations (Grigg & Kirshner, 2015; Somaweera et al., 2020). The trophic niche of crocodilians is influenced by several factors, including: ontogeny, as hatchlings feed primarily on aquatic and terrestrial invertebrate prey, juveniles increment smaller fishes and vertebrates in the diet, and adults consume more large vertebrates and fishes; flood pulse seasonality due to the high proportion of invertebrates in the wet season changing to a prevalence of fishes in the dry season (Da Silveira & Magnusson, 1999; Magnusson, Da Silva & Lima, 1987; Thorbjarnarson, 1997; Thorbjarnarson, 1993); and sex due to sexual differences in habitat use and foraging area in the breeding period (Barão-Nóbrega et al., 2016; Villamarín et al., 2011). Moreover, crocodilians take advantage of artificial or disturbed waterbodies, demonstrating ecological adaptability and resilience to persist in agricultural landscapes (Borteiro et al., 2008; Marques et al., 2020b; Pereira & Colli, 2022). Therefore, crocodilians are model organisms to detect and investigate the effects of human disturbance on the food web and assess the role of intraspecific trait variation on trophic dynamics.

*Caiman crocodilus* (Alligatoridae) inhabits a variety of natural wetlands (*e.g*., rivers, creeks, ponds, lakes, swamps, and marshes) and artificial waterbodies across their geographic distribution in Brazil, which comprehends the Amazon basin, Tocantins/Araguaia basins (central Brazil), and the Parnaíba and Coreaú basins in Ceará state (northeast Brazil) (Farias et al., 2013; Velasco & Ayarzagüena, 2010). In the Araguaia basin, agriculture development impacts natural communities by replacing native vegetation with pastures and crops (Garcia et al., 2017) and changing the hydro-geomorphological dynamics by water damming, pumping, and sedimentation (Oliveira et al., 2015), implying in new ecosystems with modified habitats and landscapes to caimans occupy (Pereira & Colli, 2022). Here, we investigate the relationship of sex, ontogeny, and habitat use factors with the trophic niche of *C. crocodilus* in a human-modified landscape of the Formoso River floodplain, a sub-basin of the middle Araguaia River basin. We conducted a multiple temporal analysis using carbon and nitrogen stable isotopes from different body tissues with different incorporation rates. We predicted that the caiman isotopic niche would be influenced by (i) tissue type, reflecting changes in food resources induced by the seasonal dynamics of the floodplain; (ii) habitat, demonstrating variability in the resource type; and (iii) sex, reflecting differences in foraging behavior and habitat use. Further, we predicted that *δ*^15^N and *δ*^13^C values should vary with body size due to the ontogenetic dietary shift.

## Materials and Methods

### Study area

We conducted the study at Praia Alta farm in the municipality of Lagoa da Confusão (10°44′0.94″S, 49°51′23.66″W), Tocantins State, Brazil. The city is the most significant rice producer in the state, with 43,600 ha of irrigated rice funded by international and state programs to expand the infrastructure of irrigation projects (IBGE, 2016). The agricultural activity follows the hydrologic regime, with rice cultivated primarily from October to April, in the rainy season, and soybean or other crops from May to September, in the dry season (Oliveira et al., 2015). Portions of this text were previously published as part of a thesis (Pereira, 2021).

Lagoa da Confusão is in the Formoso River basin, a sub-basin of the middle Araguaia River basin, in the Cerrado–Amazonia ecotone (Marques et al., 2020a). The vegetation comprises alluvial and semi-deciduous forests and floodplain grasslands (Valente, Latrubesse & Ferreira, 2013). In the wet season, the flood pulse can raise the water level to 8 m and span about 90,000 km^2^, interconnecting most waterbodies and lasting up to 5 months (Valente, Latrubesse & Ferreira, 2013). The mean annual temperature is 26 °C, the mean annual precipitation is 1,700 mm, and the air relative humidity is 40% in the dry and 90% in the wet seasons (Da Rocha et al., 2009; Valente, Latrubesse & Ferreira, 2013).

### Sampling

We sampled caimans in four habitats in July 2016: (i) river–the Formoso River, a tributary of the Javaés River, ca. 70 m wide, 5 m deep, and surrounded by riparian vegetation; (ii) lake–the Retiro Lake, covering ca. 5 ha, surrounded by riparian vegetation, and used for cattle watering; (iii) pond–a muddy water reservoir (0.3 ha, 1 m deep) for cattle watering, surrounded by pastures and with aquatic macrophytes; and (iv) ditch–bared irrigation channels (3 m wide, ca. 1.5 m deep) for rice and soybeans according to seasonality. In that period (dry season), the habitats are isolated and distributed across the agricultural landscape, but they interconnect during the wet season. Such hydrologic dynamic facilitates caimans to make seasonal and ontogenetic movements among terrestrial and aquatic habitats, with recorded mean distances <10 km over 1 year (Campos et al., 2006; Ouboter & Nanhoe, 1988), but they have small home ranges estimated between 0.048 and 3.5 km^2^ (Campos et al., 2006; Caut et al., 2019; Marioni et al., 2022; Marques et al., 2020b; Ouboter & Nanhoe, 1988). Moreover, July probably coincides with the early period of reproduction (mating period) of crocodilians in central Brazil, which is poorly known (Andrade & Coutinho, 2009), but in Amazonia the nesting period occurs in September-December (Marioni, Von Muhlen & Da Silveira, 2007; Villamarín et al., 2011).

We captured 42 caimans in nocturnal spotlight surveys using locking cable snares or by hand (Brien & Manolis, 2016; Fitzgerald, 2012). We took animals of all sizes, comprising 14 individuals in the river, seven in the lake, 13 in the ditch, and eight in the pond. We physically restrained the mouth and limbs of captured animals with ropes and adhesive tape and brought them to a field lab (Brien & Manolis, 2016). Within 24 h, from each captured caiman, we recorded the snout-vent length (SVL), body mass, and sex by cloacal examination and palpation of the penis (Reed & Tucker, 2012); collected tissue samples for SIA (below); placed a permanent and individual mark by notching tail scutes as a standardized numerical code; and released the animal at the same place of capture (Plummer & Ferner, 2012).

We also classified caimans into four size classes due to sexual dimorphism in size (Ayarzaguena, 1983; Thorbjarnarson, 1993): hatchlings (Class I = SVL < 20 cm), juveniles (Class II = 20 ≥ SVL < 60 cm), sub-adults males/adult females (Class III = 60 ≥ SVL < 90 cm), and adult males (Class IV = SVL ≥ 90 cm). Males and females achieve sexual maturity at different sizes, with Class III comprehending reproductively active females (SVL > 60 cm), sub-adult males, and small adult males (maturity from SVL > 70 cm), while Class IV consists of mature males (Guerrero et al., 2003; Souza et al., 2010; Thorbjarnarson, 1994). We conducted this study under permit SISBIO #13324-6, issued by Instituto Chico Mendes de Conservação da Biodiversidade—ICMBio, and CEUA-UnB #33786/2016, issued by Comissão de Ética no Uso de Animais da Universidade de Brasília.

### Stable isotope analyses

From each captured caiman, we collected samples of the terminal claw (5 mm) on the same finger in all animals, tail scute (1 cm^2^) from the crest region, tail muscle (2 cm^2^), and blood (3 ml) using non-lethal sampling techniques and following standard protocols (Beaupre et al., 2004; Campbell, 2015; Fleming & Fontenot, 2015). We thinly diced claw and scute samples with scissors. We collected blood from the dorsal cervical sinus using a 4 ml BD Vacutainer^®^ blood collection kit with lithium heparin, which has no significant isotopic effect on plasma and RBC samples within 3 h (Kim & Koch, 2012). In this interim, we used a centrifuge (OMEGA Mod. 1 Labor Import®) to separate samples into red blood cells (RBC) and plasma components at 3,000 rpm for 60 s. We kept all tissue samples at −80 °C in a cryogenic liquid nitrogen container. Back at the university, we washed claw, scute, and muscle samples with a 2:1 ratio chloroform: methanol solvent to extract lipids (Post et al., 2007), dried them at 50 °C to constant mass and ground them to a fine powder using mortar and pestles. We freeze-dried plasma and RBC samples for 24 h and homogenized them. All samples weighed about 1–2 mg and were stored in 3 × 5 mm tin capsules.

The carbon and nitrogen isotope ratios were determined by combustion using an elemental analyzer (CHN-1100; Carlo Erba, Emmendingen, Germany) coupled to a Thermo Finnigan Delta Plus mass spectrometer at the Isotope Ecology Lab of the Centro de Energia Nuclear na Agricultura (CENA/Universidade de São Paulo), Piracicaba, SP, Brazil. We expressed results in delta notation (*δ*), in parts per thousand (‰), based on internationally recognized standards. We used the following equation: *δ*^13^C and *δ*^15^N (‰) = (R_sample_ − R_standard_)/R_standard_ × 1.000, where R_sample_ and R_standard_ represent the heavy/light isotope molar ratio of the sample and standard, respectively. The standard used for carbon analysis was Vienna Pee Dee Belemnite (Vienna PDB; ^13^C: ^12^C ratio = 0.01118), and the standard used for nitrogen analysis was atmospheric air (^15^N: ^14^N ratio = 0.0036765). Internal standards (tropical soil and sugarcane leaves) are routinely interspersed with target samples during analysis runs. The long-term analytical error for the internal standards is 0.2‰ for both *δ*^13^C and *δ*^15^N.

Most samples had C:N ratio values within acceptable limits: plasma (mean ± SD: 3.4 ± 0.2), RBC (3.0 ± 0.2), muscle (3.4 ± 1.9), claw (3.0 ± 0.1), and scute (2.7 ± 0.1) (Post et al., 2007). However, 17 muscle samples had a C:N ratio above 4.0, indicating a high lipid content that could affect *δ*^13^C values (Logan et al., 2008; Post et al., 2007). To solve the problem, we decided to impute the *δ*^13^C values of these samples (Penone et al., 2014). We did not consider using lipid correction equations because such equations are species- and tissue-specific, focusing mainly on fishes (Logan et al., 2008).

We based the time intervals of food assimilation for each collected tissue from tissue-specific turnover rate studies with *Caiman latirostris*, a congener species of *C. crocodilus*: 90 (plasma), 190 (muscle), and 280 (RBC) days for *δ*^15^N and 80 (plasma), 130 (muscle), and 300 (RBC) days for *δ*^13^C (Caut, 2013). Claw and scute turnover rates in crocodilians are unavailable, but we considered time intervals greater than 1 year because they are metabolically inert tissues (Marques et al., 2014; Martínez del Rio et al., 2009; Vander Zanden et al., 2015). Moreover, as isotopic ratios of tissues differ from the assimilated resource due to metabolic processes, expressed as diet-tissue discrimination factors (Δ^13^C and Δ^15^N), they can affect isotopic ratios of a consumer and generate misleading inferences in between-tissue comparisons (Dalerum & Angerbjörn, 2005). Therefore, the recommendation is to add or subtract the tissue-diet discrimination value from raw isotopic tissue data to correct and conduct analysis posteriorly (*e.g*., Rosenblatt et al., 2015). However, the discrimination factors can vary with taxa, diet, and ontogeny (Caut, Angulo & Courchamp, 2009; Martínez del Rio et al., 2009; McCutchan et al., 2003; Villamarín et al., 2018), which cause effects in the isotopic niche ellipses that can generate confound interpretations (Olin et al., 2013). Using the tissue-diet discrimination values found for *Caiman latirostris* (Caut, 2013; Marques et al., 2014), we assessed the correction on our observed isotopic data and its effects on the results detailed in Supplemental Information 1. There is a strong effect of discrimination values related to diet treatment and evident limitation of experiments—large/adult animals are unexplored. Therefore, applying corrections using uncertain tissue-diet discrimination values seems arbitrary and could generate biased and misleading results than observed isotopic data (Olin et al., 2013). Here, we maintained and reported the analyses using raw isotopic values.

### Statistical analyses

We imputed 21 missing values (0.1% of all data) using the missForest package (Stekhoven & Bühlmann, 2012). They came from four *δ*^13^C and *δ*^15^N values of one individual’s plasma and RBC samples and *δ*^13^C values for muscle samples with a C:N ratio >4 of 17 individuals. Imputation is a viable solution where missing data can introduce bias and lead to incorrect conclusions due to the masking of biological patterns (Penone et al., 2014). The missForest is among the best imputation approaches for animal trait data (Penone et al., 2014; Stekhoven & Bühlmann, 2012). It is a non-parametric method that relies on random forest algorithms. Thus, a machine learning technique handles an iterative imputation scheme by training a random forest model on observed values, predicting the missing values, and proceeding iteratively (Stekhoven & Bühlmann, 2012). We assessed imputation performance using the normalized root mean squared error (NRMSE), where an excellent performance leads to a value close to 0 (Stekhoven & Bühlmann, 2012). In our case, the NRMSE was 0.023%.

Then, we evaluated the capture effort, assessing the sex ratio, size class, and habitat differences in the population structure. For that, we used a generalized linear model (GLM) with a Poisson error distribution and a log link function (Crawley, 2014), implemented in package stats (R Development Core Team, 2021). Initially, we created a full model in which the number of captured caimans was the response variable, while size class, sex, habitat, and their interactions (habitat: size class, sex: size class, and sex: habitat) were the predictors. We used an information-theory approach (Burnham & Anderson, 2002), based on the Akaike information criterion corrected for small samples (AIC_C_) to rank all possible models (including the null model) and to calculate the model-averaged coefficients, performed through package MuMIn (Bartón, 2022). Then, we found the relative predictor importance, determined as the sum of the Akaike weights (wAIC_C_) for all models containing a given predictor (Burnham & Anderson, 2002). We retained models with ΔAIC_C_ < 4.0 and wAIC_C_ > 0.1 to assess predictor coefficients. We assessed the residuals, dispersion, and outliers of best model through *simulateResiduals* function with default parameters in package DHARMa (Hartig, 2021), which showed that best model fitted and had no diagnose problems (Fig. S1).

To assess the effects of tissue, SVL, habitat, and sex on *δ*^13^C and *δ*^15^N, we used Bayesian models implemented through Integrated Nested Laplace Approximations (INLA) approach. INLA became a faster and accurate alternative for Bayesian inference than time-intensive Markov Chain Monte Carlo methods (Rue et al., 2017; Wang, Yue & Faraway, 2018). In this analysis, *δ*^13^C and *δ*^15^N values were response variables (analysis for each one); tissue, SVL, habitat, sex, and interactions (among SVL, habitat, and sex) were fixed effects; and individual (identity) were random effects to account for tissue resampling. We implemented the INLA approach using the R-INLA package (Rue, Martino & Chopin, 2009). Before, we standardized SVL around the mean with one standard deviation. For each model, we evaluated the performance based on deviance information (DIC) and Watanabe-Akaike information (WAIC) criteria (Wang, Yue & Faraway, 2018).

Next, we used the estimated Bayesian standard ellipse area metric (SEA_B_; in ‰^2^), calculated in package SIBER (Jackson et al., 2011), to assess the influence of tissue, habitat, sex, and size class factors upon isotopic niches, reflecting in a good description of intraspecific variation. We estimated the SEA_B_ containing 95% of the data through Markov chain Monte Carlo simulations with 10^4^ iterations, 10^3^ burn-ins, and two chains (Jackson et al., 2011). We did not use the corrected standard ellipse (SEA_C_), indicated for small sample size (groups for *n* < 10 observations), due to: (i) only two groups had *n* < 10; (ii) Bayesian estimates capture the same properties as SEA_C_ (Jackson et al., 2011); (iii) SEA_B_’s posterior estimates are used for between-groups comparisons; (iv) preliminarily, SEA_C_ and SEA_B_ values are strongly correlated in our data (*r* = 0.99, *R*^*2*^ = 0.99, *n* = 15), thus more advantageous uses Bayesian estimate by propagating uncertainty respect to sampling process and returning posterior distribution of the niche estimates for robust statistical comparisons.

We evaluated three niche metrics based on Hammerschlag-Peyer et al. (2011): (i) niche width, a proxy for resource variety, estimated by SEA_B_; (ii) niche position, a proxy for resource type in the exploration of the carbon and nitrogen pools, delineated by niche ellipses from Bayesian simulations in the bi-dimensional isotopic space; and (iii) niche overlap, a proxy for the degree of niche similarity and partitioning in resource and habitat use, measured through the area of overlap between two ellipses in the posterior distributions of Bayesian simulations using function *bayesianOverlap* with 95% of the data.

Variations in the niche position can indicate resource types from different foraging areas that vary in trophic structure, basal energy source, or both. Consequently, niche overlap can reflect the sharing of isotopic pools under temporal (by tissue group) and foraging areas (by habitat group) or intensity of competitive forces (between sexes). Finally, variations in the niche width can reflect variability in resource exploitation driven by factors such as resource diversity, foraging strategy, or competitive forces acting differently among groups. To compare niche width between groups (*e.g*., tissue *vs* tissue, habitat *vs* habitat, or male *vs* female), we performed pairwise tests using SEA_B_ values drawn in the simulations for each group and calculating the probability that one group was larger (reference group) than the other: SEA_B–groupA_ > SEA_B–groupB_ (Jackson et al., 2011). Thus, the probability of difference can range from 0.5 (equal probabilities or lower certainty) to 1.00 (higher certainty). We performed all statistical tests in R (R Development Core Team, 2021).

## Results

The GLM analysis revealed that body size class was the most important predictor of variations in the population structure (Table 1). The sampled population was predominantly composed of individuals in Class II (*n* = 19) and Class III (*n* = 20) compared to other sizes (Class I: *n* = 1; and Class IV: *n* = 2). The sex ratio was balanced, with 1.1 females for each male (males: *n* = 20; females: *n* = 22), with no statistical significance as indicated by model-averaged coefficients. No significant model included habitat. Overall, the analysis revealed that our sample consisted primarily of individuals of medium size, well-balanced across sexes and habitats.

**Table 1:** Factors affecting the number of captures of *Caiman crocodilus* at Lagoa da Confusão, Tocantins state, Brazil. The generalized linear models assumed a Poisson error distribution. *df*, degrees of freedom; AIC_C_, Akaike information criterion adjusted for small samples; ΔAIC_C_, difference in AIC_C_ between candidate and best model (minimum AIC_C_); wAIC_C_, Akaike weight that represents normalized likelihood or weight of evidence in favor of candidate model; SE, standard error; *z*, value in *z*-distribution. The dataset consisted of 210 values from five tissue of 42 caimans.

Model selection	*df*	AIC_C_	ΔAIC_C_	wAIC_C_
Size class	4	82.4	0	0.723
Size class + Sex	5	85.1	2.73	0.185
Size class + Habitat	7	88.0	5.60	0.044
Size class + Sex + Size class: Sex	8	88.2	5.78	0.040
Size class + Habitat + Sex	8	91.5	9.10	0.008
Size class + Habitat + Sex + Size class: Sex	11	97.5	15.14	0
Size class + Habitat + Sex + Habitat: Sex	11	102.8	20.45	0
(Null model)	1	112	29.63	0
Size class + Habitat + Sex + Size class: Sex + Habitat: Sex	14	113.4	31.05	0
Sex	2	114.2	31.81	0
Habitat	4	115.8	33.39	0
Habitat	5	118.5	36.13	0
Size class + Habitat + Size class: Habitat	16	125	42.62	0
Habitat	8	126.9	44.48	0
Size class + Habitat + Sex + Size class: Habitat	17	134.4	51.97	0
Size class + Habitat + Sex + Size class: Habitat + Size class: Sex	20	166.1	83.72	0
Size class + Habitat + Sex + Size class: Habitat + Habitat: Sex	20	171.4	89.02	0
Size class + Habitat + Sex + Size class: Habitat + Size class: Sex + Habitat: Sex	23	232.3	149.88	0

Model-averaged coefficients	Estimate	SE	z	*P*
(Intercept)	−2.070	1.002	1.975	0.048
Size class (Class II)	2.944	1.026	2.745	0.006
Size class (Class III)	2.996	1.025	2.796	0.005
Size class (Class IV)	0.693	1.225	0.541	0.588
Sex (Male)	−0.095	0.309	0.295	0.768

Relative variable importance	Size class	Sex
Importance	1.00	0.20

### Factors influencing ${{\mathrm{\delta }}^{{\mathrm{13}}}}{\mathrm{C}}$^13^C and ${{\mathrm{\delta }}^{{\mathrm{15}}}}{\mathrm{N}}$^15^N

**Figure 1:**
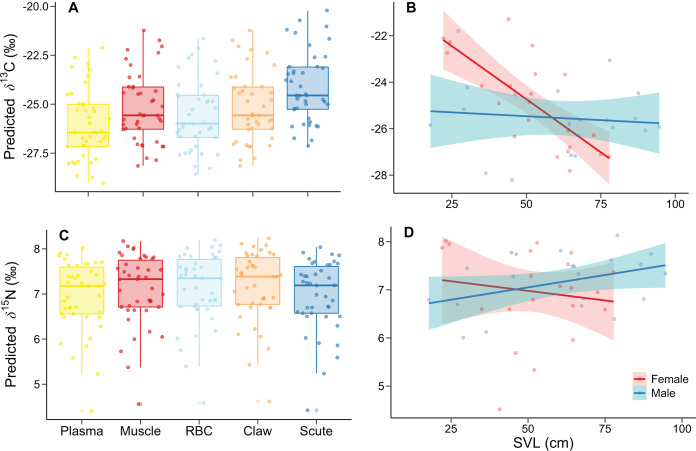
Relationship of *δ*^13^C and *δ*^15^N with (A, C) tissue and (B, D) the sex-SVL interaction of *Caiman crocodilus* at Lagoa da Confusão, Tocantins state, Brazil. Tissues are ordered according to turnover rates (Caut, 2013). Except for (A) and (C), each point corresponds to the mean isotopic value for all tissues of one individual. Lines indicate the model fit, and their respective polygons correspond to the 95% confidence interval. The dataset consisted of 210 values from five tissue of the 42 caimans.

The best model for *δ*^13^C had much lower DIC (551.00) and WAIC (554.53) values than the intercept-only model (DIC = 899.16; WAIC = 899.11). INLA modeling revealed differences in tissue, SVL, and the sex-SVL interaction (Table 2). *δ*^13^C values increased according to isotopic incorporation time, from smaller plasma to higher scute values (Fig. 1A). There was a decrease of *δ*^13^C values with SVL; however, this relationship depended on sex, with a more substantial *δ*^13^C decrease in females than males (Fig. 1B). The remaining predictors included zero in their 95% credible intervals of posterior distributions, indicating lower association probabilities with *δ*^13^C values (Table 2).

**Table 2:** Factors affecting the concentration of *δ*^13^C in *Caiman crocodilus* at Lagoa da Confusão, Tocantins state, Brazil. Values represent posterior estimates (mean, SD, and 95% credibility interval) from Bayesian models relating tissue, habitat, sex, snout-vent length (SVL), and their interactions to *δ*^13^C values of *Caiman crocodilus*. The dataset consisted of 210 values from five tissue of 42 caimans.

Predictor	Mean	SD	Q_0.025_	Q_0.975_
Intercept	−25.101	0.578	−26.243	−23.960
Tissue (muscle)	0.000	0.176	−0.345	0.345
Tissue (plasma)	−0.876	0.176	−1.222	−0.531
Tissue (red blood cells)	−0.418	0.176	−0.763	−0.073
Tissue (scute)	1.019	0.176	0.674	1.364
Habitat (lake)	0.010	0.827	−1.627	1.644
Habitat (pond)	2.782	1.919	−1.020	6.566
Habitat (river)	−0.486	0.852	−2.172	1.195
Sex (male)	−0.023	0.807	−1.621	1.571
SVL	−1.705	0.479	−2.651	−0.760
Habitat (lake): Sex (male)	−1.627	1.385	−4.366	1.110
Habitat (pond): Sex (male)	−1.588	2.118	−5.769	2.601
Habitat (river): Sex (male)	−1.778	1.268	−4.285	0.728
Habitat (lake): SVL	0.320	0.922	−1.503	2.140
Habitat (pond): SVL	4.302	3.280	−2.196	10.766
Habitat (river): SVL	−1.902	2.025	−5.904	2.099
Sex (male): SVL	1.530	0.711	0.122	2.934
Habitat (lake): Sex (male): SVL	0.980	1.415	−1.818	3.774
Habitat (pond): Sex (male): SVL	−4.405	3.372	−11.059	2.263
Habitat (river): Sex (male): SVL	3.215	2.175	−1.087	7.507

For the *δ*^15^N, the best model had much lower DIC (272.86) and WAIC (276.74) values than the intercept-only model (DIC = 550.78; WAIC = 551.25). We found differences in tissue, SVL, and the sex-SVL interaction (Table 3). Tissues differed by ~0.2‰, with lower and higher predicted *δ*^15^N in plasma and claw, respectively (Fig. 1C). *δ*^15^N related negatively with SVL, likely due to female trends (the reference group), while males differed with a positive *δ*^15^N–SVL relationship (Fig. 1D). The remaining predictors included zero in their 95% credible intervals of posterior distributions, indicating lower association probabilities with *δ*^15^N values (Table 3).

**Table 3:** Factors affecting the concentration of *δ*^15^N in *Caiman crocodilus* at Lagoa da Confusão, Tocantins state, Brazil. Values represent posterior estimates (mean, SD, and 95% credibility interval) from Bayesian models relating tissue, habitat, sex, snout-vent length (SVL), and their interactions to *δ*^15^N values of *Caiman crocodilus*. Parameter estimates that differ from zero in the 95% credibility interval reflect intercept differences. The dataset consisted of 210 values from five tissue of 42 caimans.

Predictor	Mean	SD	Q_0.025_	Q_0.975_
Intercept	7.198	0.250	6.705	7.691
Tissue (muscle)	−0.058	0.091	−0.237	0.120
Tissue (plasma)	−0.209	0.091	−0.388	−0.031
Tissue (red blood cells)	−0.037	0.091	−0.215	0.141
Tissue (scute)	−0.196	0.091	−0.375	−0.018
Habitat (lake)	−0.053	0.354	−0.754	0.647
Habitat (pond)	−1.259	0.831	−2.902	0.381
Habitat (river)	0.490	0.365	−0.232	1.211
Sex (male)	0.583	0.346	−0.101	1.267
SVL	−0.485	0.205	−0.890	−0.080
Habitat (lake): Sex (male)	−0.778	0.594	−1.952	0.394
Habitat (pond): Sex (male)	0.079	0.916	−1.732	1.888
Habitat (river): Sex (male)	−0.966	0.544	−2.041	0.108
Habitat (lake): SVL	0.521	0.395	−0.260	1.301
Habitat (pond): SVL	1.063	1.421	−1.747	3.869
Habitat (river): SVL	−0.732	0.870	−2.452	0.986
Sex (male): SVL	0.827	0.305	0.224	1.429
Habitat (lake): Sex (male): SVL	−0.129	0.606	−1.328	1.069
Habitat (pond): Sex (male): SVL	−1.323	1.461	−4.211	1.562
Habitat (river): Sex (male): SVL	0.461	0.934	−1.386	2.306

### Effects on isotopic niche parameters of caimans

**Figure 2:**
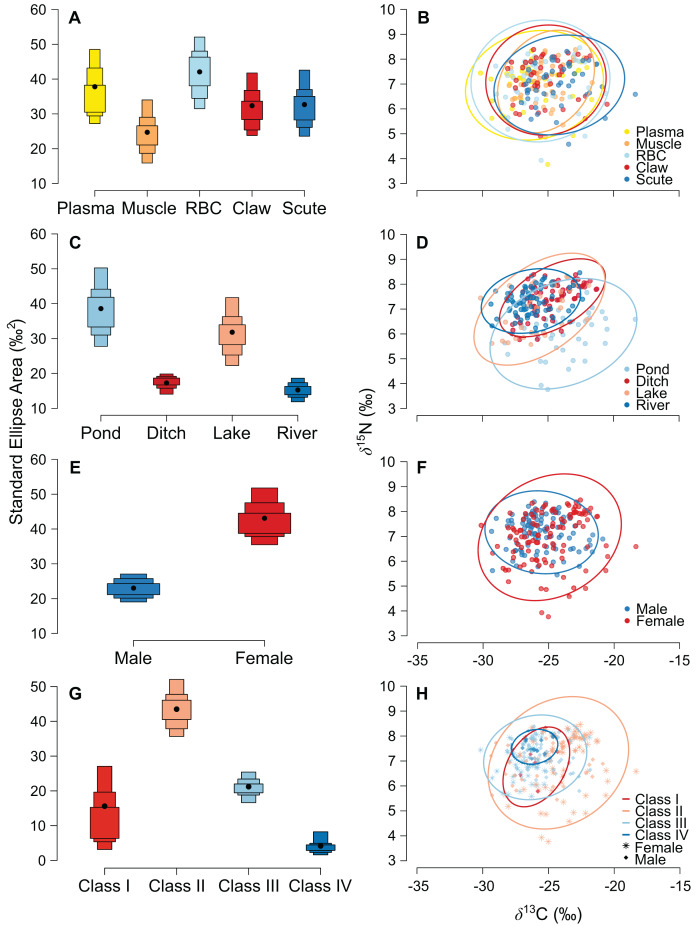
Estimated niche width and isotopic niches for (A, B) tissues, (C, D) habitats, (E, F) sexes, and (G, H) size classes of *Caiman crocodilus* at Lagoa da Confusão, Tocantins state, Brazil. Scatter plots for habitats and sexes exhibit mean isotopic values of all tissues from each individual. Solid lines represent the core isotopic niche space. Black dots correspond to the mean, and boxes represent 50%, 75%, and 95% credibility intervals.

**Figure 3:**
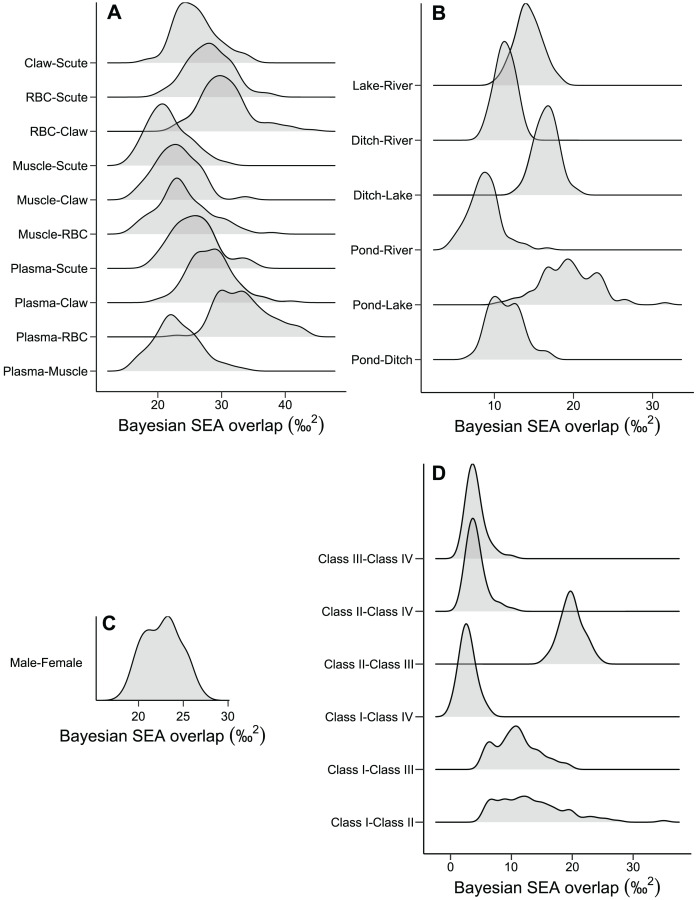
Density distributions of the niche overlap area for pairwise comparisons from Bayesian simulations of the niche ellipses according to (A) tissue, (B) habitat, (C) sex, and (D) size classes.

The niche width differed among tissues in ascending order: muscle, claw, scute, plasma, and RBC (Fig. 2A and Table S1). The tissues were concentrated in a specific region of the isotopic niche, with high overlap overall (Fig. 2B). Muscle–scute ellipses had the smallest niche overlap, while RBC–plasma had higher overlaps (Fig. 3A).

The pond and lake caimans had broader niches than the river and ditch (Fig. 2C and Table S1). Isotopic niches were also clustered by habitat, except caimans in the pond with a broader niche and a short displacement in location (Fig. 2D). The pattern of niche overlap varied in the habitat comparisons (Fig. 3B). Patterns of niche width and overlap between habitats within tissues overall resembled patterns of the pooled tissues, except for muscle (Fig. S2).

There was a pronounced difference in niche width between sexes, with females having a larger niche that fully encompassed the males’ niche (Figs. 2E and 2F; Fig. 3C). Overall, patterns of niche metrics between sexes within habitats (Fig. S3) or tissues (Fig. S4) resembled the pooled data. Additionally, the niche width varied with size class, with juveniles (Class II) standing out with a larger niche, while sub-adult and adult animals (Class III and IV) had smaller niches (Fig. 2G). Juveniles’ niches encompassed other class niches (Fig. 2H and Table S1). Otherwise, Class IV’s niche was restricted to a smaller isotopic region, included in other niche ellipses. However, we found low niche overlap in general, with higher overlaps driven by Class II and III (Fig. 3D). Considering the size classes by sex, females maintained larger niche widths than males in the Classes II and III, with short displacement in isotopic position (Figs. S5A and S5B). Across habitats, the niche width varied among size classes (Figs. S5C, S5E, S5G and S5I), with some differences from all data (Fig. S5A), due to dataset subdivisions that increased uncertainty in the estimates. In some habitats, sexes of the same size class occupied partially distinct isotopic regions (lake and river for Class II, and ditch for Class III; Figs. S5D, S5F and S5J), and size classes differed partially or totally in the same sex in two situations—Classes II and III for males in the lake and females in the ditch (Figs. S5F and S5J).

## Discussion

We assessed patterns of variation in the trophic niche of *C. crocodilus* in an agricultural landscape of the Formoso River floodplain. The isotopic niche metrics effectively characterized and revealed intraspecific variation in caimans in the floodplain. We found that the isotopic niche is relatively constant through time but highly variable among habitats considering the niche width, regardless of their origin (natural or anthropogenic). Moreover, we found significant effects of sex and ontogeny. Our findings present relevant factors and mechanisms that reduce intraspecific competition and promote coexistence, enabling caiman populations to thrive in human-modified landscapes.

### Little temporal variability of the tissue niches

The Bayesian modeling revealed differences among tissues for both isotopes. While the differences in *δ*^13^C were greater than 1‰ among some tissues, *δ*^15^N showed differences that were not larger than the analytical error, demonstrating slight variation among tissues (a null ecological difference). However, the consistent use of the carbon and nitrogen pools in the isotopic space (*i.e*., tissue niche overlaps) indicated little temporal variability, suggesting assimilation of the same resource mixture with no apparent seasonal variation. Our results differ from studies with fishes and macrophytes that detected seasonal differences in isotopic patterns (Sepúlveda-Lozada et al., 2017;Wantzen, Fellerhoff & Voss, 2010). Further, other studies revealed seasonal differences in the proportions of prey categories used by *C. crocodilus* (Da Silveira & Magnusson, 1999; Magnusson, Da Silva & Lima, 1987; Thorbjarnarson, 1993). Although we did not conduct gut content analyses, the long-term isotopic consistency demonstrates the whole possible isotopic space by caimans from available resource exploitation in the floodplain. Therefore, caiman isotopic niches are also conditioned to isotopic variability of resource mixture, which can have no or minimal differences across time and space (Bearhop et al., 2004; Layman et al., 2007a; Yeakel et al., 2016).

Crocodilians show slower tissue incorporation rates than other groups (Caut, 2013; Rosenblatt & Heithaus, 2013). Some crocodilian tissues can record extended periods of the dietary information, with the slowest rates enabling the integration of short and long-term isotopic data (Dalerum & Angerbjörn, 2005). The niche width variability can associate the frequency of temporal dietary changes to tissue incorporation rates (Yeakel et al., 2016). Thus, the peak variance should occur during dietary shifts, evidenced in tissues that capture such isotopic changes—in our case, RBC with assimilation throughout the year (wet and dry season). Moreover, the isotopic variability decreases when the tissue incorporation rate (*i.e*., longer turnover time) is higher than the timescale of dietary switching. For instance, the claw and scute tissues depicted a similar physiological process, averaging the isotopic variability due to the slowest incorporation rates. A smaller niche width (*e.g*., muscle) could occur in the wet season due to lower environmental temperatures, shortage of nutritious resources, and lower feeding opportunities that induce a period of reduced foraging activity and food intake (Da Silveira & Magnusson, 1999; Magnusson, Da Silva & Lima, 1987; Thorbjarnarson, 1993). In combination, these conditions could produce physiological effects, including slower digestion and assimilation rates through low metabolic demand of the ectotherm lifestyle (Grigg & Kirshner, 2015; Lang, 1987).

However, we recognize that the sensitivity of diet-tissue discrimination factors imposes limitations in interpreting our results. As detailed in the Supplemental Information 1, the diet-tissue discrimination values vary according to feed in controlled dietary studies, possibly confounding interpretations (Caut, Angulo & Courchamp, 2009). In the case of wild animals, the prey diversity or mixture could have different or imprecise values from the literature. Beyond diet (protein quality), it is widely acknowledged the sensibility of discrimination values to reproductive and nutritional status, tissue, age, and sex (Caut, Angulo & Courchamp, 2009; Kurle et al., 2014; Lecomte et al., 2011; Martínez del Rio et al., 2009). Lecomte et al. (2011) indicated that sex and age could concomitantly and synergically affect isotopic discrimination by acting in a tissue-, diet-, and isotope-specific manner in the Arctic fox (*Vulpes lagopus*). Villamarín et al. (2018) found no relationship between trophic position estimates from dietary-based information and stable isotope analysis (*δ*^15^N values) in crocodilians, arguing that discrimination values can vary ontogenetically. Since diet quality influences the discrimination factor, discrimination values should be greater in older individuals than juveniles due to protein content increases along the lifespan of crocodilians (Villamarín et al., 2018). Additionally, Scharnweber et al. (2021) found a relationship between the standard metabolic rate (SMR) and trophic discrimination factor for carbon and nitrogen in the muscle of Eurasian perch (*Perca fluviatilis*); as SMR varies inversely with body size, discrimination values vary as well. Therefore, controlled experiments with crocodilians aimed at determining the diet-tissue discrimination factors are limited when restricted to the juvenile population and adults are missing (Caut, 2013; Hanson et al., 2015; Rosenblatt & Heithaus, 2013), providing unappropriated values to other size classes. Recognizing that different discrimination values can affect niche ellipses of a species (Olin et al., 2013), we did not apply corrections from diet-tissue discrimination factors. Our study is not aimed at reconstructing the diet of *Caiman crocodilus*, but at assessing differences in isotopic niche parameters that can be associated with intraspecific variation.

### Disentangling isotopic niches from habitat suitability and caiman movements

Cross-ecosystem studies revealed significant differences in crocodilian trophic niches as resources differ clearly in diversity and isotopic composition (Adame et al., 2018; Hanson et al., 2015; Nifong, Layman & Silliman, 2015; Rosenblatt & Heithaus, 2011). Moreover, crocodilian species also exhibit clear niche divergence under sympatry (Villamarín et al., 2017). In agreement with the Bayesian modeling results, niche redundancy among habitats revealed by niche position indicates that caimans use resources from diverse habitat isotopic pools. However, habitats had differences in food resources, reflecting in two distinct groups of caiman niche width, possibly linked to variation in their suitability.

Habitat suitability to caimans can result from productivity, connectivity, and caiman population behavior. First, some smaller waterbodies may not supply the energy demands of this top predator. Thus, caimans may expand their foraging areas to complement the energy demand (allochthonous subsidy), exploiting terrestrial resources (Adame et al., 2018; Jardine et al., 2017; Villamarín et al., 2017). Caimans in the artificial pond can increase their niche width through this mechanism, where they may reside in this aquatic habitat but not participate in its food web (Adame et al., 2018; Jardine et al., 2017). Second, habitat heterogeneity, proximity, and between-habitat movements can diversify the resources exploited by transient caimans in a population, increasing the niche width. These factors suggest a plausible explanation for the caimans in the lake, where lake-river movements happen quickly. Third, ditch and river habitats can form a single population, with transient individuals using similar foraging strategies, whereas ditches are just a shelter habitat with scarce resources to exploit. In these habitats, caimans may have a different feeding strategy than other habitats, for instance, by developing some degree of individual specialization (Araújo, Bolnick & Layman, 2011; Bolnick et al., 2011) since habitat type is a driver due to resource availability (Rosenblatt et al., 2015). The smaller niche width of caimans from the ditch may reflect a lower resource diversity resulting from the biotic homogenization in the agricultural system (Burdon, McIntosh & Harding, 2020; Layman et al., 2007b). Nevertheless, caimans can move and use more suitable adjacent habitats (like rivers and lakes) to cope with the cost of disturbance. The high niche overlap between them supports this explanation. In this sense, landscape configuration and composition influence how populations use habitats and explore the resources across the landscape, dynamizing trophic relations of the food web and affecting their trophic niche measures (Rooney, McCann & Moore, 2008; Swihart et al., 2006).

Anthropogenic disturbance and human-managed landscapes can affect the trophic niches by promoting distinct isotopic compositions than natural habitats for biodiversity and increasing niche overlap (Burdon, McIntosh & Harding, 2020; Carvalho et al., 2015; Manlick & Pauli, 2020; Zambrano, Valiente & Vander Zanden, 2010). Our results mimic previous studies in tropical natural floodplains, with energy sources deriving primarily from autochthonous aquatic resources (Caut et al., 2019; Villamarín et al., 2017). Nonetheless, with the expansion of the agricultural frontier in central Brazil, energy derived from crops or pastures can become increasingly important in the food webs (Carvalho et al., 2015; Wantzen, Fellerhoff & Voss, 2010). The occurrence of caimans in agricultural ditches and artificial ponds can suggest the incorporation of agricultural energy inputs, providing crop-derived carbon and affecting the energy flux and trophic relations in the whole food web (Pereira, 2021). Pereira & Colli (2022) reported higher caiman body conditions in anthropogenic habitats, suggesting they favor foraging strategies and agricultural energy intake.

### Sexual body-size dimorphism triggering isotopic niche partitioning

Caimans have a well-documented ontogenetic dietary shift (Da Silveira & Magnusson, 1999; Magnusson, Da Silva & Lima, 1987; Thorbjarnarson, 1993). There is a significant change in the prey types and sizes with increasing body size in crocodilians (Grigg & Kirshner, 2015; Radloff, Hobson & Leslie, 2012; Thorbjarnarson, 1993)—for instance, from 85 g in hatchlings to 20 kg in our study, but with some conventional dietary studies reporting a high niche overlap of prey items among size classes (through prey diversity indexes) that indicates some degree of intraspecific competition (Soria-Ortiz, Charruau & Reynoso, 2020). Alternatively, isotopic studies show niche divergence through ontogenetic variation along stable isotopes or in the isotopic niches (Adame et al., 2018; Caut et al., 2019; Marques et al., 2013; Radloff, Hobson & Leslie, 2012). Corroborating with those studies, we found sex-related ontogenetic shifts in both isotopes, reflecting the changes and differences in the basal energy sources and trophic levels. Such differences suggest that sexual body-size dimorphism can trigger niche divergence and decrease intrasexual competition (Nifong, Layman & Silliman, 2015; Radloff, Hobson & Leslie, 2012). However, Villamarín et al. (2018) suggest caution on the *δ*^15^N results (trophic position), arguing that trophic discrimination factors are ontogeny-dependent; therefore, a unique trophic discrimination factor can influence the *δ*^15^N values and cause misinterpretation about the ontogeny-trophic position relationship through stable isotope analysis. Such level of information is still lacking, remaining for future controlled isotopic studies reveal the discrimination factor values according to ontogeny, tissues, and their physiological and ecological background mechanisms.

Distinct isotopic niches between size classes, in general, were not apparent, but we found clear differences between sexes or size classes in some habitats (see Fig. S5). Further, juveniles demonstrated remarkable plasticity by occupying a substantial isotopic niche space. Considering that food type intake relates to prey available as the habitat and season (Magnusson, Da Silva & Lima, 1987; Soria-Ortiz, Charruau & Reynoso, 2020; Thorbjarnarson, 1993), differences in the isotopic niches of size classes indicate that caimans are partitioning food resources with distinct isotopic composition on a habitat or microhabitat scale (Marques et al., 2013). Factors of hierarchical social organization and agonistic behaviors driven by body size can influence movements and home ranges in crocodilians (Grigg & Kirshner, 2015). Priority of access to food resources and mates can drive male territoriality (Campbell et al., 2013; Caut et al., 2019; Marioni et al., 2022; Marques et al., 2020b). Dominant adult males can have site fidelity to maximize their reproductive success with territories that overlap females’ home ranges or take advantage of the abundant resources. In contrast, expelled animals (*e.g*., weak males) consist of subordinate and nomad caimans that are forced to cover larger areas to mate or feed to avoid dangerous conflicts.

On the one hand, the isotopic niche of males in our results can consist of animals with site residency and lower prey diversity consumption, resulting in smaller niches that overlap with the female’s niche, like in river and ditch (see Fig. S5). On the other hand, the subordinate animals (ontogenetic and sexual groups) change the home range size, prey diversity, and habitats, increasing the opportunity to exploit different resources. This results in trophic niche partitioning between habitats (Adame et al., 2018; Caut et al., 2019; Marques et al., 2013). Crocodilian studies reported a variation in movement according to body size (Campbell et al., 2013; Campos et al., 2006; Marioni et al., 2022), where juveniles move longer distances than adults or *vice-versa*. *Caiman crocodilus* is considered a sedentary species, with short movements (<5 km) and a small home range, <24 ha (Ouboter & Nanhoe, 1988), compared to other South American caimans (Campos et al., 2006; Caut et al., 2019; Marioni et al., 2022; Marques et al., 2020b). Under that perspective, a broader niche of juveniles than other size classes in our study can be a response from such synergic ecological forces, in which they may constitute more transient and opportunistic individuals with wider home ranges than other size classes. Otherwise, our misbalance in the capture effort likely reduced our inference of isotopic niche parameters for Classes I and IV. Such classes had one hatchling and two animals (only adult males), respectively, which we sampled in only one habitat. By reverberating, the male animals can also be underrepresented. Indeed, those classes could differ in their isotopic niches from our results because of isotopic variation related to the diversity of habitats, available prey, and different home ranges.

Additionally, the reproduction period can promote sexual niche divergence (Beck et al., 2007; Lima et al., 2019; Smith et al., 2015). In *Caiman crocodilus*, prior conventional dietary studies reported no divergence between sexes (Da Silveira & Magnusson, 1999; Thorbjarnarson, 1993), but new information associated with reproduction and nesting behavior brought evidence of sexual and seasonal dietary differences (Barão-Nóbrega et al., 2016). During the breeding season, females can increase their movements to find suitable nesting habitats (Campbell et al., 2013). During this time, nesting females can ingest more terrestrial resources, including under nesting attendance behavior (Barão-Nóbrega et al., 2016). Meanwhile, immature females and males consume aquatic resources in waterbodies. In this sense, nesting females can differ in the isotopic niche of the immature females and males related to the consumption of terrestrial prey with a different basal source. In our study, females had a larger niche than males, though they occupied the same isotopic region overall. However, we found partially distinct in isotopic niches between sex in some habitats (*e.g*., in ditch and river; Fig. S5). Likely, the female population consists of mature and immature individuals with distinct movement and foraging strategies, resulting in a broader isotopic niche. However, we found no effects of reproduction on habitat and resource use through isotopic niche parameters. In part, this result stems from the fact that we could not assess the reproductive status of females. Our findings highlight sexual differences in caiman foraging ecology, which could ultimately promote reduced intraspecific competition.

## Conclusions

Species’ sexual and body size/age traits vary in the population in their ecological niches to promote coexistence and reduced competition, translating into habitat segregation and foraging strategies in a spatiotemporal dynamic. Our results suggest that seasonal variation in the resource use across the Formoso River floodplain is not evident through isotopic niche parameters, indicating a little temporal variability in the basal energy source and trophic level of the food web. Due to habitat suitability, connectivity, and movements, patterns of caiman resource use are similar among habitats. Considering that our study did not focus on achieving diet reconstruction information of *Caiman crocodilus*, there were limitations in finding the mechanisms that trigger the niche overlap across habitats and tissues. Future directions for researchers to elucidate the mechanisms underlying niche overlap in dietary studies in the Formoso River floodplain, identifying the isotopic composition of prey types and determining their contribution to the caiman diet together with complementary gut content analysis. Otherwise, we found niche divergence related to sex and ontogeny, with a particular trophic dynamic driven by females’ and juveniles’ foraging strategies to reduce intraspecific competition. Given the population dynamic of those groups, it is necessary to consider surrounding habitats and landscape heterogeneity of the Formoso River floodplain in the sustainable management and conservation actions, even artificial waterbodies through restoration and maintenance, which are conditioned to the protection of areas for caiman reproduction and foraging.

## Supplemental Information

10.7717/peerj.15915/supp-1 Supplemental Information 1 Comparative evaluation of isotopic data correction through tissue-diet discrimination factor application on raw isotopic tissue data of *Caiman crocodilus*..

10.7717/peerj.15915/supp-2 Supplemental Information 2 Supplemental tables and figures.

## Additional Information and Declarations

### Field Study Permissions

The following information was supplied relating to field study approvals (*i.e*., approving body and any reference numbers): Field experiments were approved by the Instituto Chico Mendes de Conservação da Biodiversidade—ICMBio (permit SISBIO #13324-6)

### Data Availability

The following information was supplied regarding data availability: The data is available at Mendeley: Pereira, André; Nardoto, Gabriela; Colli, Guarino (2023), “Data for: Sources of intraspecific variation in the isotopic niche of a semi-aquatic predator in a human-modified landscape,” Mendeley Data, V1, DOI 10.17632/6dfzrxcsjz.1.
