# Supplementary material for: Sources of intraspecific variation in the isotopic niche of a semi-aquatic predator in a human-modified landscape
Source: PeerJ. 2023 Aug 30;11:e15915. doi: 10.7717/peerj.15915 (PMC10474837; doi:10.7717/peerj.15915)
Supplement: Supplemental Information 1 [file peerj-11-15915-s001.pdf]

PeerJ - Life and Environment

Supplemental Information 1

Sources of intraspecific variation in the isotopic niche of a semi-aquatic top predator in a human-modified landscape

André Costa Pereira[1,*], Gabriela Bielefeld Nardoto[2], Guarino Rinaldi Colli[1]

[1] Departamento de Zoologia, Instituto de Ciências Biológicas, Universidade de Brasília, Brasília, Distrito Federal, Brasil.
[2] Departamento de Ecologia, Instituto de Ciências Biológicas, Universidade de Brasília, Brasília, Distrito Federal, Brasil.

* Corresponding author
E-mail: rancoper@gmail.com

ORCID:
ACP (ORCID: 0000-0003-4432-7163)
GBN (ORCID: 0000-0001-8062-7417)
GRC (ORCID: 0000-0002-2628-5652)

Application of tissue-diet discrimination factors in the analyses

We used the values of tissue-diet discrimination factors for carbon ($\Delta^{13}C$) and nitrogen ($\Delta^{15}N$) from studies with *Caiman latirostris* (Caut 2013; Marques et al. 2014), a congener species of *Caiman crocodilus*, which reported values for each collected tissues in our study (Table 1). As the tissue-diet discrimination values in Caut's study differ according with diet, we analyzed and reported separately the effects upon *Caiman crocodilus* isotopic niches considering three treatments: (a) observed isotopic data, (b) corrections by discrimination factor using chicken diet ($\Delta^{13}C_{Chicken}$ and $\Delta^{15}N_{Chicken}$), and (c) corrections by discrimination factor using Roach fish diet ($\Delta^{13}C_{Fish}$ and $\Delta^{15}N_{Fish}$). We applied the same $\Delta^{13}C$ and $\Delta^{15}N$ values for claw and scute in both corrections.

We evaluated comparatively among treatments the niche metrics (position, overlap, and width) considering tissue, habitat, and sex factors. Detailed niche metric estimations are in the Materials and Methods of main text.

Results

The isotopic niches among tissues for each treatment had different results in the niche position: a isotopic concentration and high niche overlap in the observed data (Figure 1A); a isotopic concentration and high niche overlap, but with a specific displacement for muscle niche using the $\Delta^{13}C_{Chicken}$ and $\Delta^{15}N_{Chicken}$ correction (Figure 1B); and a descending change of isotopic niche along $\delta^{15}N$ axis (Figure 1C), metabolically active tissues situating in high $\delta^{15}N$ values, while inert tissues situating in low $\delta^{15}N$ values when applied the $\Delta^{13}C_{Fish}$ and $\Delta^{15}N_{Fish}$ correction. Consequently, the niche overlap changed in the tissue pairwise comparisons from observed data to treatment-fish correction (Figure 1D-F). Otherwise, the niche widths showed identical results (Figure 1G-I).

For isotopic niches among habitats, the correction treatments had broader isotopic distribution, but with similar configuration in the niche position compared to observed data (Figure 2A-C). The niche overlap in habitat pairwise comparisons increased with treatments (Figure 2D-F). Otherwise, the niche width showed similar results, but with increase in the SEA_B (Figure 2G-I).

For the sex factor, the results followed the habitat result pattern: broader isotopic distribution and similar configuration in the niche position with correction treatments (Figure 3A-C); the niche overlap between sexes increased with treatments (Figure 3D-F); and similar results in the niche width, but SEA_B increased with treatment (Figure 3G-I).

Discussion

This exploration showed possible misleading results for tissue factor in the isotopic niche metrics when applied distinct values of the discrimination factors. Regarding to habitat and sex factors, the results from correction treatments maintained similar compared to observed isotopic data. With obvious effect on tissue factor, the treatments had similar results in the niche width, whereas the niche position and overlap changed drastically driven by increase of range in the δ^{15}N axis, indicating distinct nitrogen pools according with tissues. Indeed, the differences among tissues are caused by selection of Δ^{13}C and Δ^{15}N values alone, suggesting a sensitivity and vulnerability to a bias in the results.

The variation in diet-tissue discrimination factors among tissues can differ among individuals in a population due to life stage, reproductive, or nutritional status, but intrinsically relates to tissue factors, as macromolecule compositions, protein turnover, amino acid allocation (Kurle et al. 2014; Martínez del Rio et al. 2009; McCutchan Jr et al. 2003; Vanderklift & Ponsard 2003). However, diet has pivotal influence due to protein quality and content that directly increase or decrease the Δ^{15}N (Caut et al. 2009; Kurle et al. 2014; Martinez del Rio & Wolf 2005; Martínez del Rio et al. 2009; Whiteman et al. 2021). So, we pointed out relevant aspects to consider about experiments. First, to produce Δ^{13}C and Δ^{15}N values, controlled experiments use specific diets that is not realistic compared to natural food webs and resource diversification that varies in the diet quality (content and nutrition propriety) for a consumer. Second, animals that have dietary ontogenetic shifts with increase of protein acquisition as the body size could vary the discrimination values ontogenetically, like crocodilians (Villamarín et al. 2018), and thus, implying in another variation factor to account regardless tissue type. Third, controlled experiments with crocodilians restrict analyses to juvenile population (e.g. Caut 2013; Hanson et al. 2015; Rosenblatt & Heithaus 2013), lacking adults in the sampling, reflecting a limitation of scope to apply the discrimination values compared to sampled population in ecological studies, as our case. In this sense, the application of a unique discrimination value for all body size seems unrealistic. Therefore, the selection of which discriminant values to use in the analyses seems equivocated and arbitrary, remaining elusive and uncertain in the corrections.

References

Caut S. 2013. Isotope incorporation in broad-snouted caimans (crocodilians). *Biology Open* 2:629-634. DOI: 10.1242/bio.20134945

Caut S, Angulo E, Courchamp F. 2009. Variation in discrimination factors ($\Delta^{15}N$ and $\Delta^{13}C$): the effect of diet isotopic values and applications for diet reconstruction. *Journal of Applied Ecology* 46:443-453. DOI: 10.1111/j.1365-2664.2009.01620.x

Hanson JO, Salisbury SW, Campbell HA, Dwyer RG, Jardine TD, Franklin CE. 2015. Feeding across the food web: The interaction between diet, movement and body size in estuarine crocodiles (*Crocodylus porosus*). *Austral Ecology* 40:275-286. DOI: 10.1111/aec.12212

Kurle CM, Koch PL, Tershy BR, Croll DA. 2014. The effects of sex, tissue type, and dietary components on stable isotope discrimination factors ($\Delta^{13}C$ and $\Delta^{15}N$) in mammalian omnivores. *Isotopes in Environmental and Health Studies* 50:307-321. DOI: 10.1080/10256016.2014.908872

Marques TS, Bassetti LAB, Lara NRF, Araujo MS, Piña CI, Camargo PB, Verdade LM. 2014. Isotopic discrimination factors ($\Delta^{15}N$ and $\Delta^{13}C$) between tissues and diet of the broad-snouted caiman (*Caiman latirostris*). *Journal of Herpetology* 48:332-337. DOI: 10.1670/12-274

Martinez del Rio C, Wolf BO. 2005. Mass balance models for animal isotopic ecology. In: Starck MA, Wang T, eds. *Physiological and ecological adaptations to feeding in vertebrates*. Enfield, NH: Science Publishers, 141-174.

Martínez del Rio C, Wolf N, Carleton SA, Gannes LZ. 2009. Isotopic ecology ten years after a call for more laboratory experiments. *Biological Reviews* 84:91-111. DOI: 10.1111/j.1469-185X.2008.00064.x

McCutchan Jr JH, Lewis Jr WM, Kendall C, McGrath CC. 2003. Variation in trophic shift for stable isotope ratios of carbon, nitrogen, and sulfur. *Oikos* 102:378-390. DOI: 10.1034/j.1600-0706.2003.12098.x

Rosenblatt AE, Heithaus MR. 2013. Slow isotope turnover rates and low discrimination values in the American alligator: Implications for interpretation of ectotherm stable isotope data. *Physiological and Biochemical Zoology* 86:137-148. DOI: 10.1086/668295

Vanderklift MA, Ponsard S. 2003. Sources of variation in consumer-diet $\delta^{15}N$ enrichment: a meta-analysis. *Oecologia* 136:169-182. DOI: 10.1007/s00442-003-1270-z

Villamarín F, Jardine TD, Bunn SE, Marioni B, Magnusson WE. 2018. Body size is more important than diet in determining stable-isotope estimates of trophic position in crocodilians. *Scientific Reports* 8:1-11. DOI: 10.1038/s41598-018-19918-6

Whiteman JP, Rodriguez Curras M, Feeser KL, Newsome SD. 2021. Dietary protein content and digestibility influences discrimination of amino acid nitrogen isotope values in a terrestrial omnivorous mammal. *Rapid Communications in Mass Spectrometry* 35:e9073. DOI: 10.1002/rcm.9073

Table 1. Reference values of the tissue-diet discrimination factors for carbon ($\Delta^{13}C$) and nitrogen ($\Delta^{15}N$) for collected tissues.

Tissue	Diet	$\Delta^{13}C$ (‰)	$\Delta^{15}N$ (‰)	Reference
Plasma	Chicken	-0.08	0.08	Caut (2013)
	Roach fish	-0.11	-2.24	Caut (2013)
Muscle	Chicken	-0.04	-1.59	Caut (2013)
	Roach fish	1.06	-2.50	Caut (2013)
RBC	Chicken	-0.52	0.39	Caut (2013)
	Roach fish	0.66	0.93	Caut (2013)
Claw	Chicken	1.2*	1.1*	Marques et al. (2014)
Scute	Chicken	0.9*	0.9*	Marques et al. (2014)

RBC: red blood cells; * Mean values

Figure 1. Isotopic niches, density distributions of the niche overlap area for pairwise comparisons from Bayesian simulations of the niche ellipses, and estimated niche width for tissue group in each treatment: A, D,and G from observed isotopic data; B, E, and H from corrections by tissue-diet discrimination factors using chicken diet ($\Delta^{13}C_{Chicken}$ and $\Delta^{15}N_{Chicken}$); C, F, and I from corrections by tissue-diet discrimination factors using Roach fish diet ($\Delta^{13}C_{Fish}$ and $\Delta^{15}N_{Fish}$). In the scatter plots, solid lines represent the core isotopic niche space. Black dots correspond to the mean and boxes represent the 50%, 75% and 95% credible intervals.

Figure 2. Isotopic niches, density distributions of the niche overlap area for pairwise comparisons from Bayesian simulations of the niche ellipses, and estimated niche width for habitat group in each treatment: A, D,and G from observed isotopic data; B, E, and H from corrections by tissue-diet discrimination factors using chicken diet ($\Delta^{13}C_{Chicken}$ and $\Delta^{15}N_{Chicken}$); C, F, and I from corrections by tissue-diet discrimination factors using Roach fish diet ($\Delta^{13}C_{Fish}$ and $\Delta^{15}N_{Fish}$). Scatter plots exhibit the mean isotopic values of all tissues from each individual. Solid lines represent the core isotopic niche space. Black dots correspond to the mean and boxes represent the 50%, 75% and 95% credible intervals.

Figure 3. Isotopic niches, density distributions of the niche overlap area for pairwise comparisons from Bayesian simulations of the niche ellipses, and estimated niche width for sex group in each treatment: A, D,and G from observed isotopic data; B, E, and H from corrections by tissue-diet discrimination factors using chicken diet ($\Delta^{13}C_{Chicken}$ and $\Delta^{15}N_{Chicken}$); C, F, and I from corrections by tissue-diet discrimination factors using Roach fish diet ($\Delta^{13}C_{Fish}$ and $\Delta^{15}N_{Fish}$). Scatter plots exhibit the mean isotopic values of all tissues from each individual. Solid lines represent the core isotopic niche space. Black dots correspond to the mean and boxes represent the 50%, 75% and 95% credible intervals.

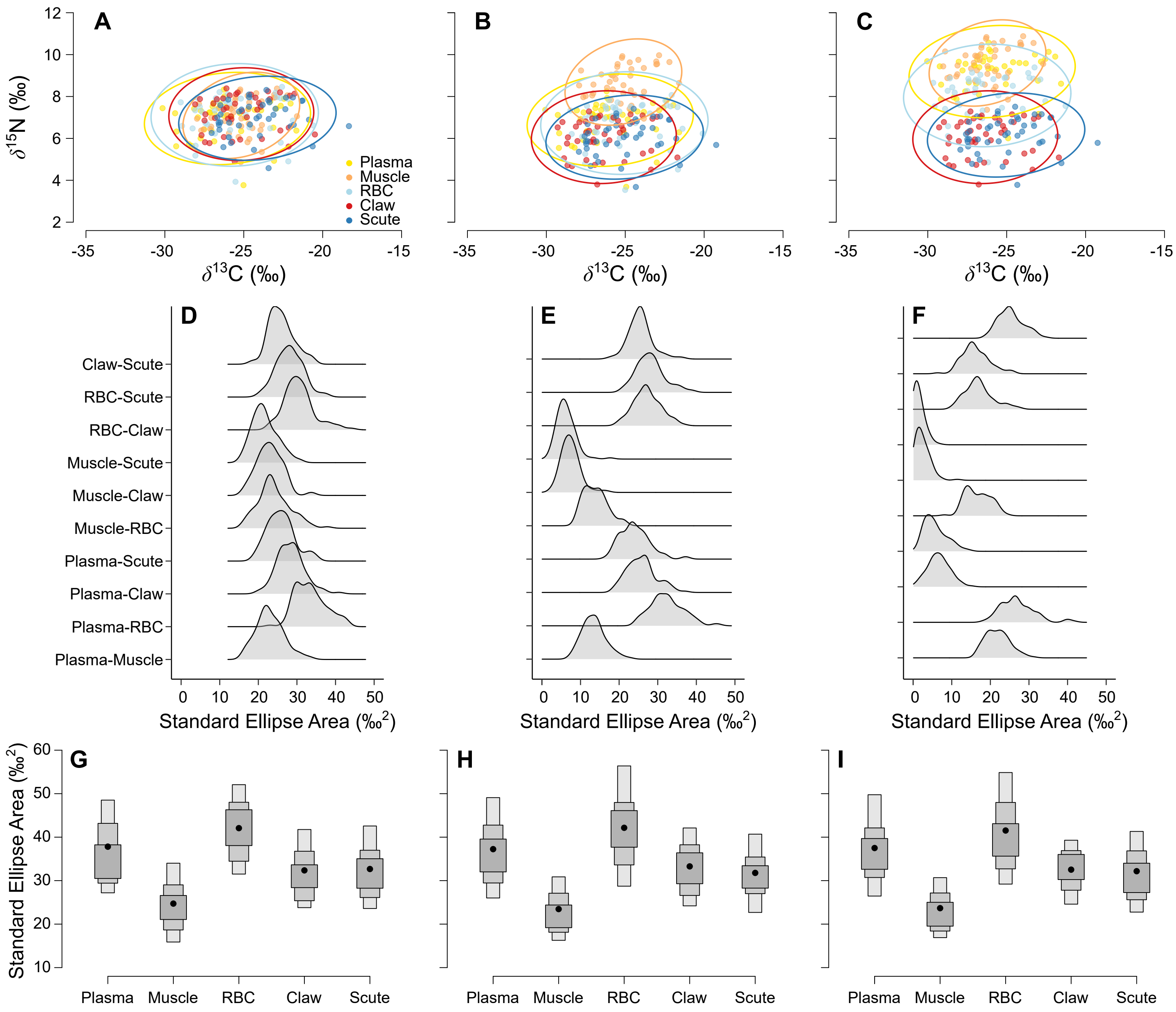

A
B
C
D
E
F
G
H
I
Plasma
Muscle
RBC
Claw
Scute
δ15N (‰)
δ13C (‰)
Claw-Scute
RBC-Scute
RBC-Claw
Muscle-Scute
Muscle-Claw
Muscle-RBC
Plasma-Scute
Plasma-Claw
Plasma-RBC
Plasma-Muscle
Standard Ellipse Area (‰2)

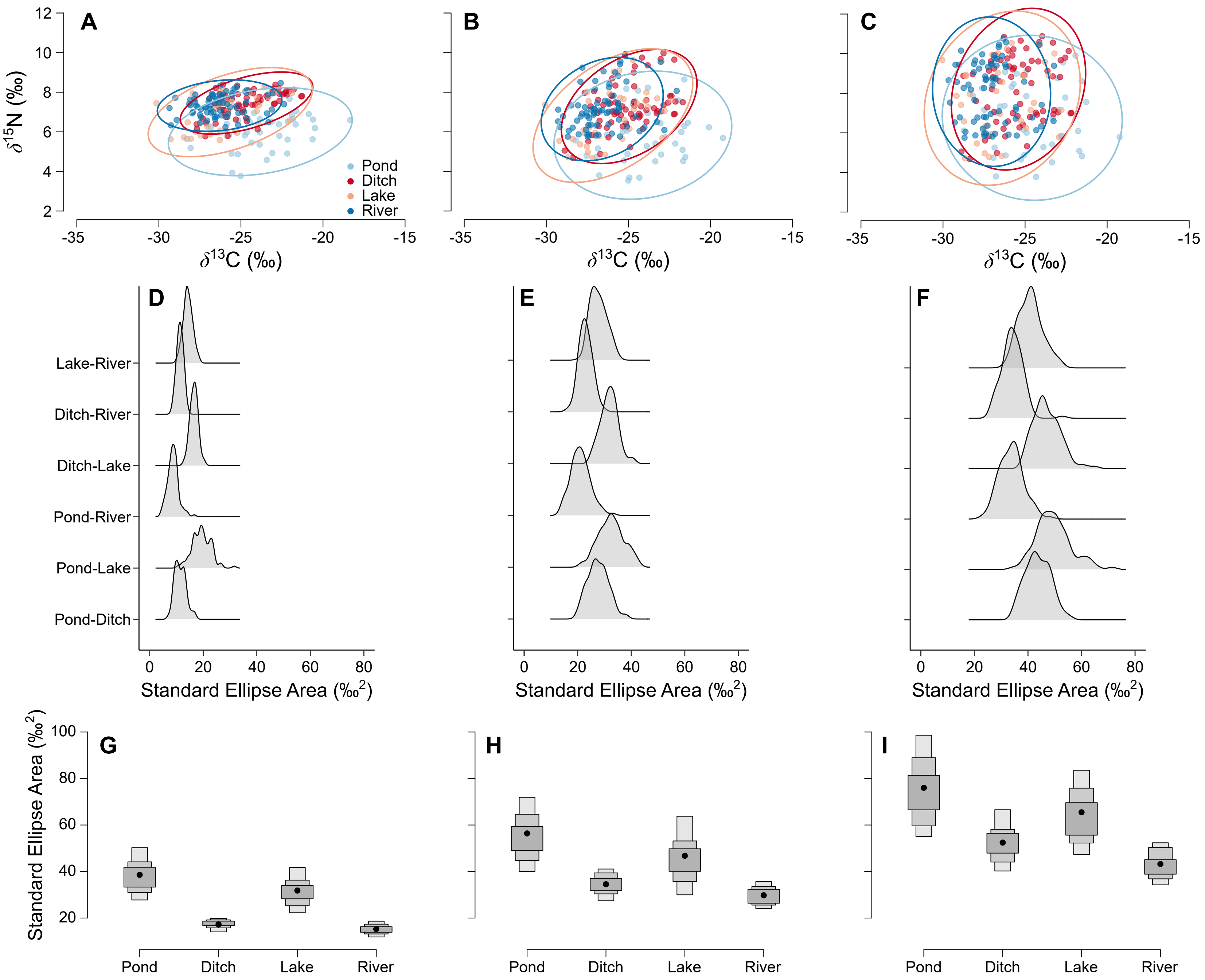

A
B
C
δ15N (‰)
δ13C (‰)
Pond
Ditch
Lake
River
D
E
F
Lake-River
Ditch-River
Ditch-Lake
Pond-River
Pond-Lake
Pond-Ditch
Standard Ellipse Area (‰2)
G
H
I
Standard Ellipse Area (‰2)
Pond
Ditch
Lake
River

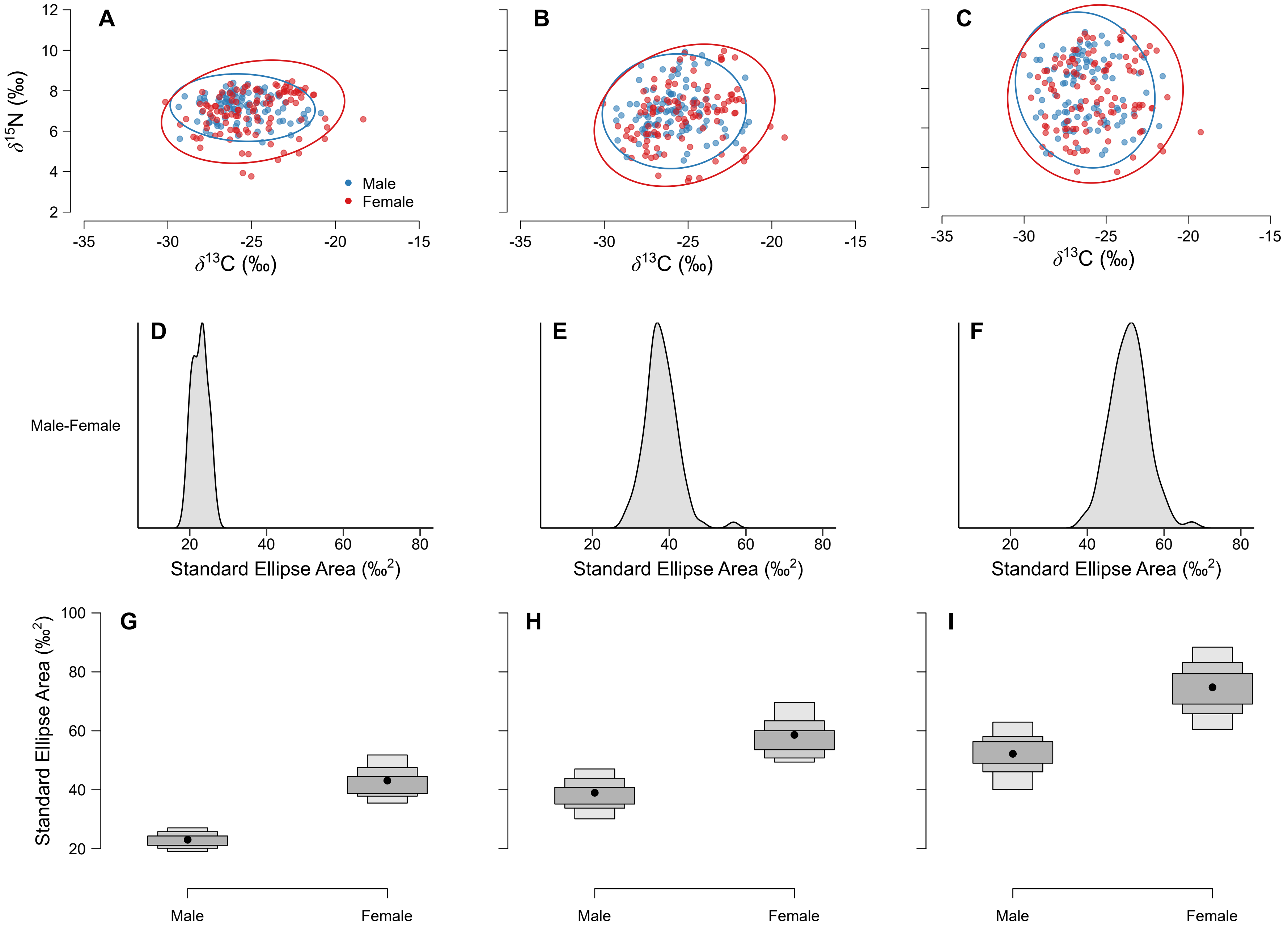

A
B
C
δ^{15}N (‰)
12
10
8
6
4
2
-35
-30
-25
-20
-15
δ^{13}C (‰)
Male
Female
D
E
F
Male-Female
20
40
60
80
Standard Ellipse Area (‰^{2})
G
H
I
Standard Ellipse Area (‰^{2})
100
80
60
40
20
Male
Female
