# Supplementary material for: Sources of intraspecific variation in the isotopic niche of a semi-aquatic predator in a human-modified landscape
Source: PeerJ. 2023 Aug 30;11:e15915. doi: 10.7717/peerj.15915 (PMC10474837; doi:10.7717/peerj.15915)
Supplement: Supplemental Information 2 [file peerj-11-15915-s002.pdf]

PeerJ - Life and Environment

Supplemental Information 2

Sources of intraspecific variation in the isotopic niche of a semi-aquatic top predator in a human-modified landscape

André Costa Pereira[1,*], Gabriela Bielefeld Nardoto[2], Guarino Rinaldi Colli[1]

[1] Departamento de Zoologia, Instituto de Ciências Biológicas, Universidade de Brasília, Brasília, Distrito Federal, Brasil.

[2] Departamento de Ecologia, Instituto de Ciências Biológicas, Universidade de Brasília, Brasília, Distrito Federal, Brasil.

* Corresponding author
E-mail: rancoper@gmail.com

ORCID:
ACP (ORCID: 0000-0003-4432-7163)
GBN (ORCID: 0000-0001-8062-7417)
GRC (ORCID: 0000-0002-2628-5652)

Table S1. Probability of difference between groups for tissues, habitats, and size classes. The first group was the largest group (reference for comparisons).

Tissue	
Comparison	Probability
$SEA_{B\text{-}RBC} > SEA_{B\text{-}muscle}$	0.99
$SEA_{B\text{-}plasma} > SEA_{B\text{-}muscle}$	0.98
$SEA_{B\text{-}RBC} > SEA_{B\text{-}claw}$	0.91
$SEA_{B\text{-}scute} > SEA_{B\text{-}muscle}$	0.86
$SEA_{B\text{-}claw} > SEA_{B\text{-}muscle}$	0.86
$SEA_{B\text{-}RBC} > SEA_{B\text{-}scute}$	0.85
$SEA_{B\text{-}RBC} > SEA_{B\text{-}plasma}$	0.74
$SEA_{B\text{-}plasma} > SEA_{B\text{-}claw}$	0.74
$SEA_{B\text{-}plasma} > SEA_{B\text{-}scute}$	0.72
$SEA_{B\text{-}scute} > SEA_{B\text{-}claw}$	0.54
Habitat	
$SEA_{B\text{-}pond} > SEA_{B\text{-}river}$	1.0
$SEA_{B\text{-}pond} > SEA_{B\text{-}ditch}$	1.0
$SEA_{B\text{-}lake} > SEA_{B\text{-}river}$	1.0
$SEA_{B\text{-}lake} > SEA_{B\text{-}ditch}$	1.0
$SEA_{B\text{-}pond} > SEA_{B\text{-}lake}$	0.86
$SEA_{B\text{-}ditch} > SEA_{B\text{-}river}$	0.80
Size class	
$SEA_{B\text{-}Class\ II} > SEA_{B\text{-}Class\ III}$	1.0
$SEA_{B\text{-}Class\ II} > SEA_{B\text{-}Class\ IV}$	1.0
$SEA_{B\text{-}Class\ III} > SEA_{B\text{-}Class\ IV}$	1.0
$SEA_{B\text{-}Class\ I} > SEA_{B\text{-}Class\ II}$	0.99
$SEA_{B\text{-}Class\ I} > SEA_{B\text{-}Class\ IV}$	0.99
$SEA_{B\text{-}Class\ I} > SEA_{B\text{-}Class\ III}$	0.80

Figure S1. Residual diagnoses of normality, outlier, and overdispersion of the best model in the GLM: (A) QQ plot residuals, and (B) Residuals vs. predicted residuals relationship. Normality, outlier, and overdispersion had no significance.

Figure S2. Estimated niche width and isotopic niches for habitats. Habitat data for (A, B) plasma, (C, D) muscle, (E, F) RBC, (G, H) claw, and (I, J) scute. Solid lines represent the core isotopic niche space. Black dots correspond to the mean and boxes represent the 50%, 75% and 95% credible intervals.

Figure S3. Estimated niche width and isotopic niches for sexes. Sex data in the (A, B) river, (C, D) lake, (E, F) pond, and (G, H) ditch. Scatter plots had the isotopic values from all tissues. Solid lines represent the core isotopic niche space. Black dots correspond to the mean and boxes represent the 50%, 75% and 95% credible intervals.

Figure S4. Estimated niche width and isotopic niches for sexes. Sex data for (A, B) plasma, (C, D) muscle, (E, F) RBC, (G, H) claw, and (I, J) scute. Scatter plots had the isotopic values from all habitats. Solid lines represent the core isotopic niche space. Black dots correspond to the mean and boxes represent the 50%, 75% and 95% credible intervals.

Figure S5. Estimated niche width and isotopic niches for size classes. Size class data in the (A, B) all habitats, (C, D) river, (E, F) lake, (G, H) pond, and (I, J) ditch. Colors relate to size class, while bold words comprehend female’s size classes. Scatter plots had the isotopic values from all tissues. Solid lines represent the core isotopic niche space. Black dots correspond to the mean and boxes represent the 50%, 75% and 95% credible intervals.

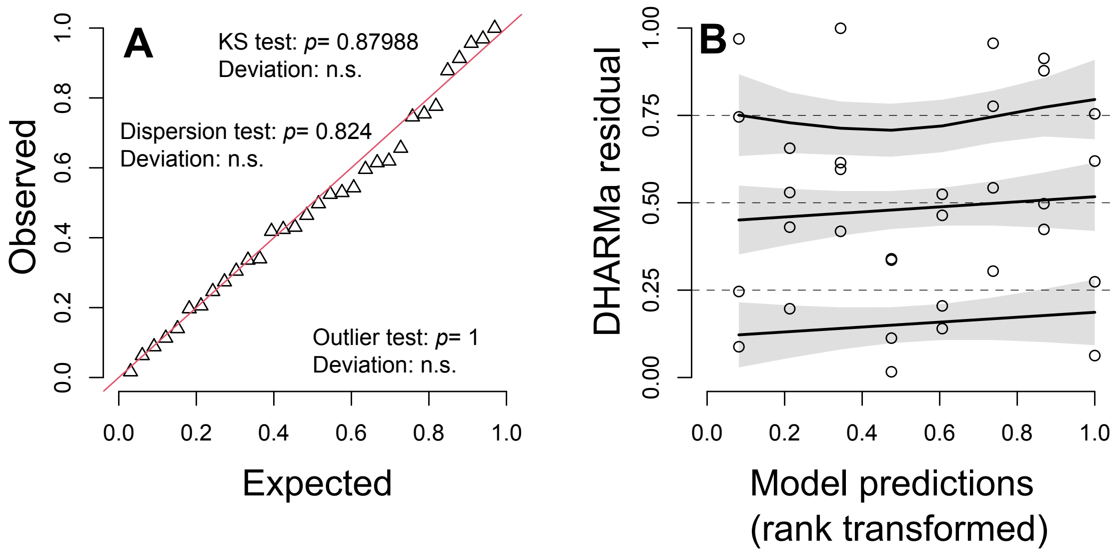
A
KS test: p= 0.87988
Deviation: n.s.
Dispersion test: p= 0.824
Deviation: n.s.
Outlier test: p= 1
Deviation: n.s.
Observed
Expected
0.0
0.2
0.4
0.6
0.8
1.0
B
DHARMa residual
0.00
0.25
0.50
0.75
1.00
Model predictions
(rank transformed)

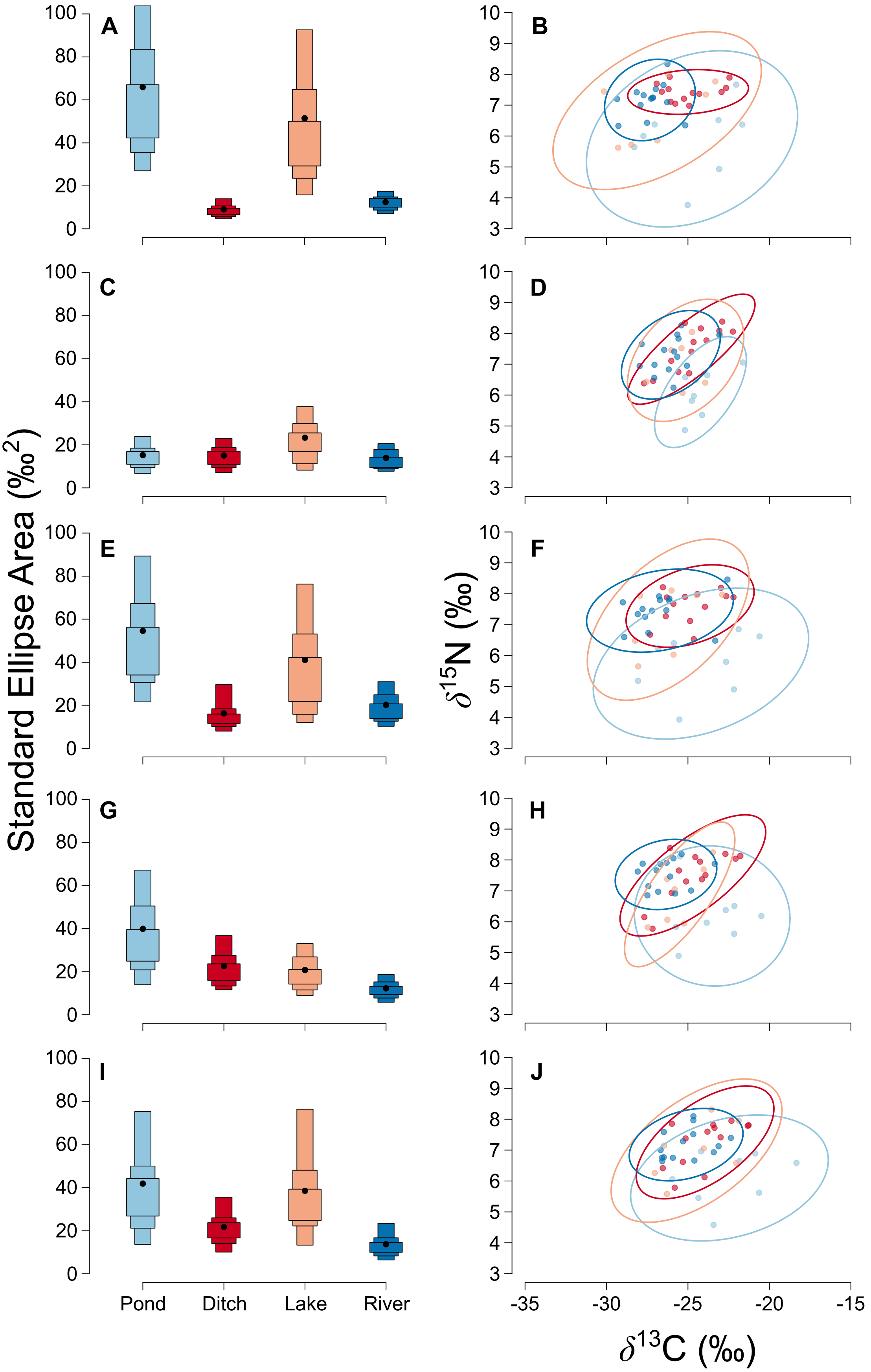

A
B
C
D
E
F
G
H
I
J
Standard Ellipse Area (‰^{2})
δ^{15}N (‰)
Pond
Ditch
Lake
River
δ^{13}C (‰)
100
80
60
40
20
0
10
9
8
7
6
5
4
3
-35
-30
-25
-20
-15

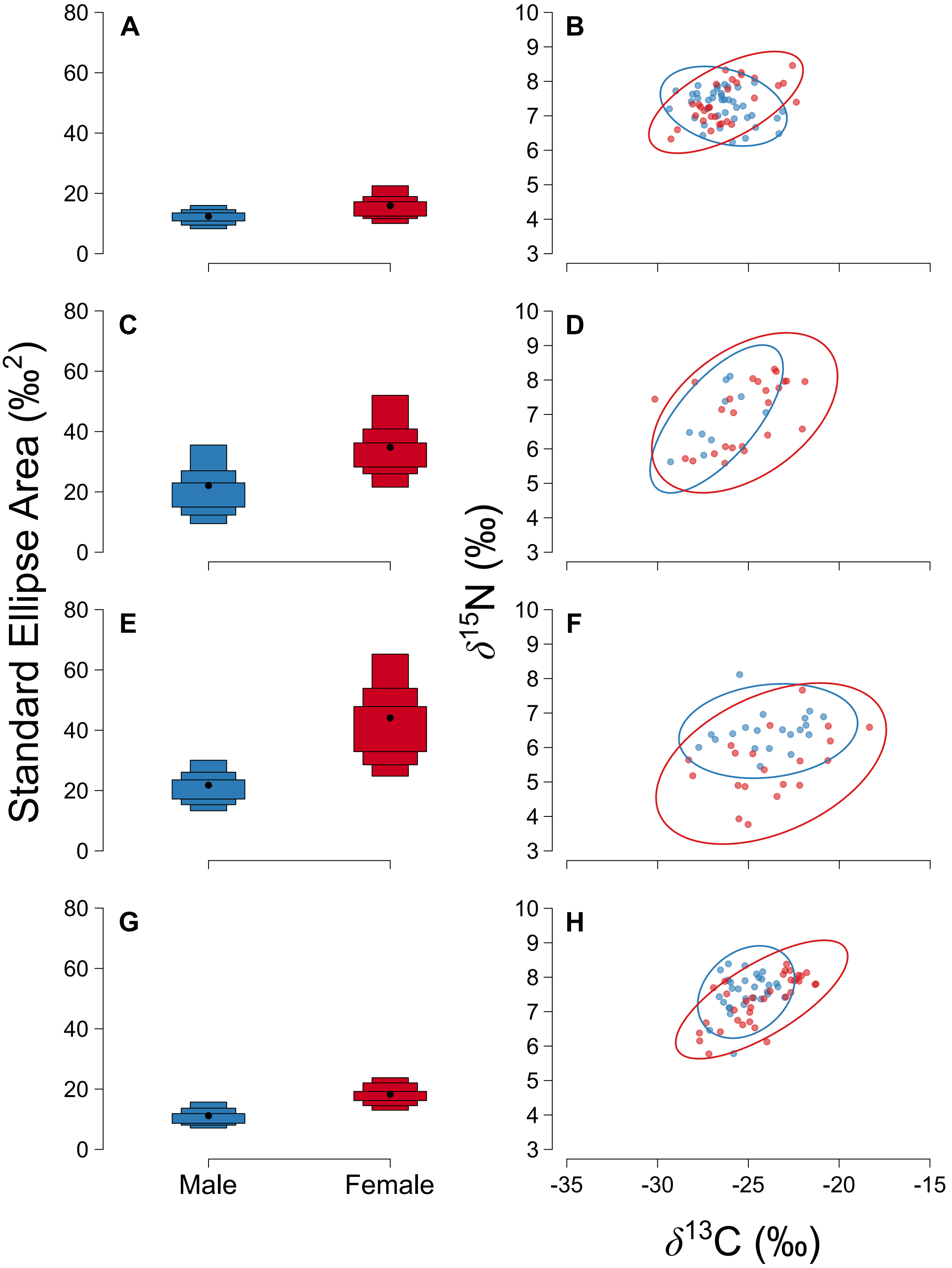

A
B
C
D
E
F
G
H
Standard Ellipse Area (‰²)
δ15N (‰)
δ13C (‰)
Male
Female
80
60
40
20
0
10
9
8
7
6
5
4
3
-35
-30
-25
-20
-15

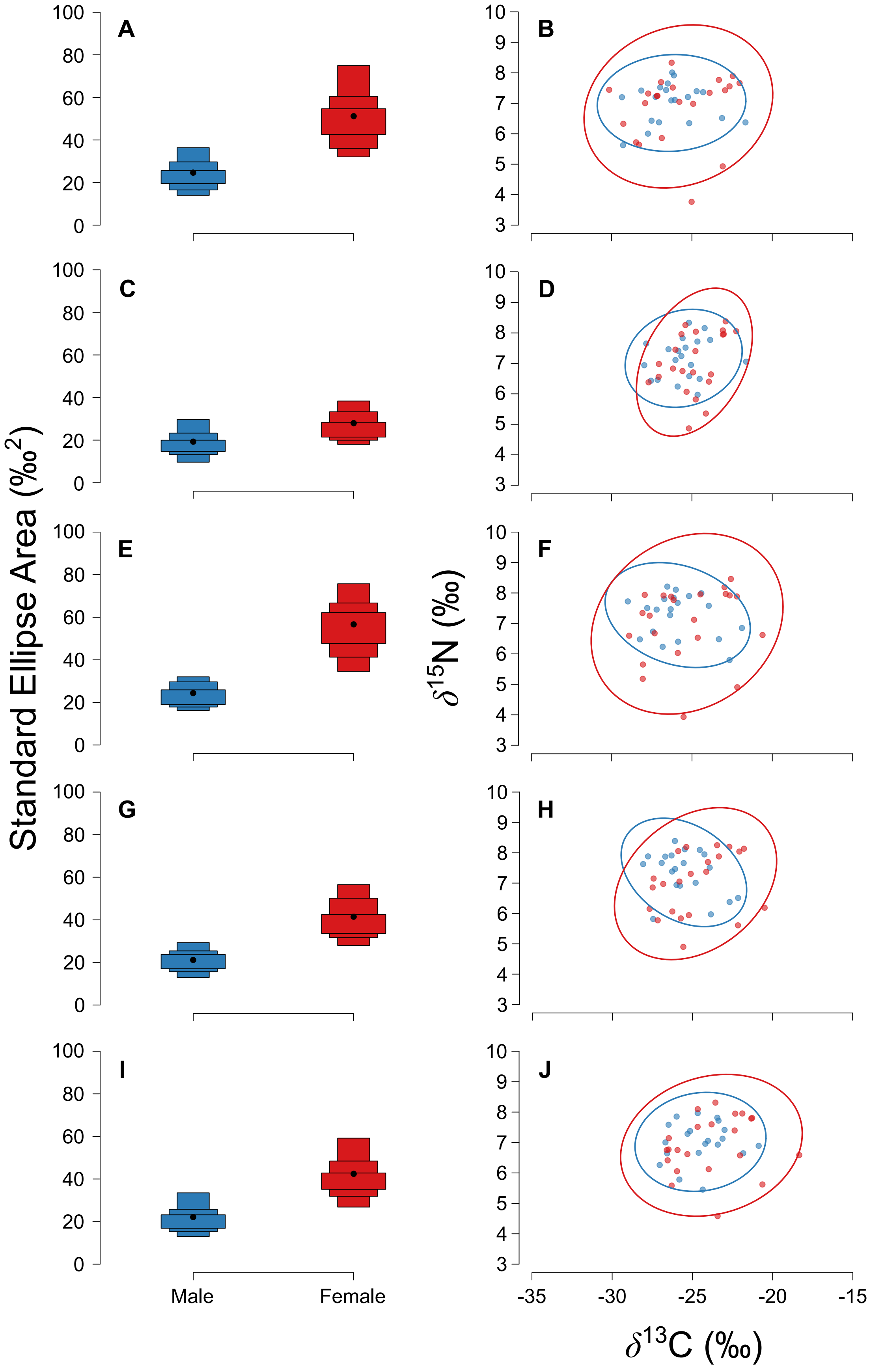

Standard Ellipse Area (‰²)
A
B
C
D
E
F
G
H
I
J
Male
Female
δ¹⁵N (‰)
δ¹³C (‰)

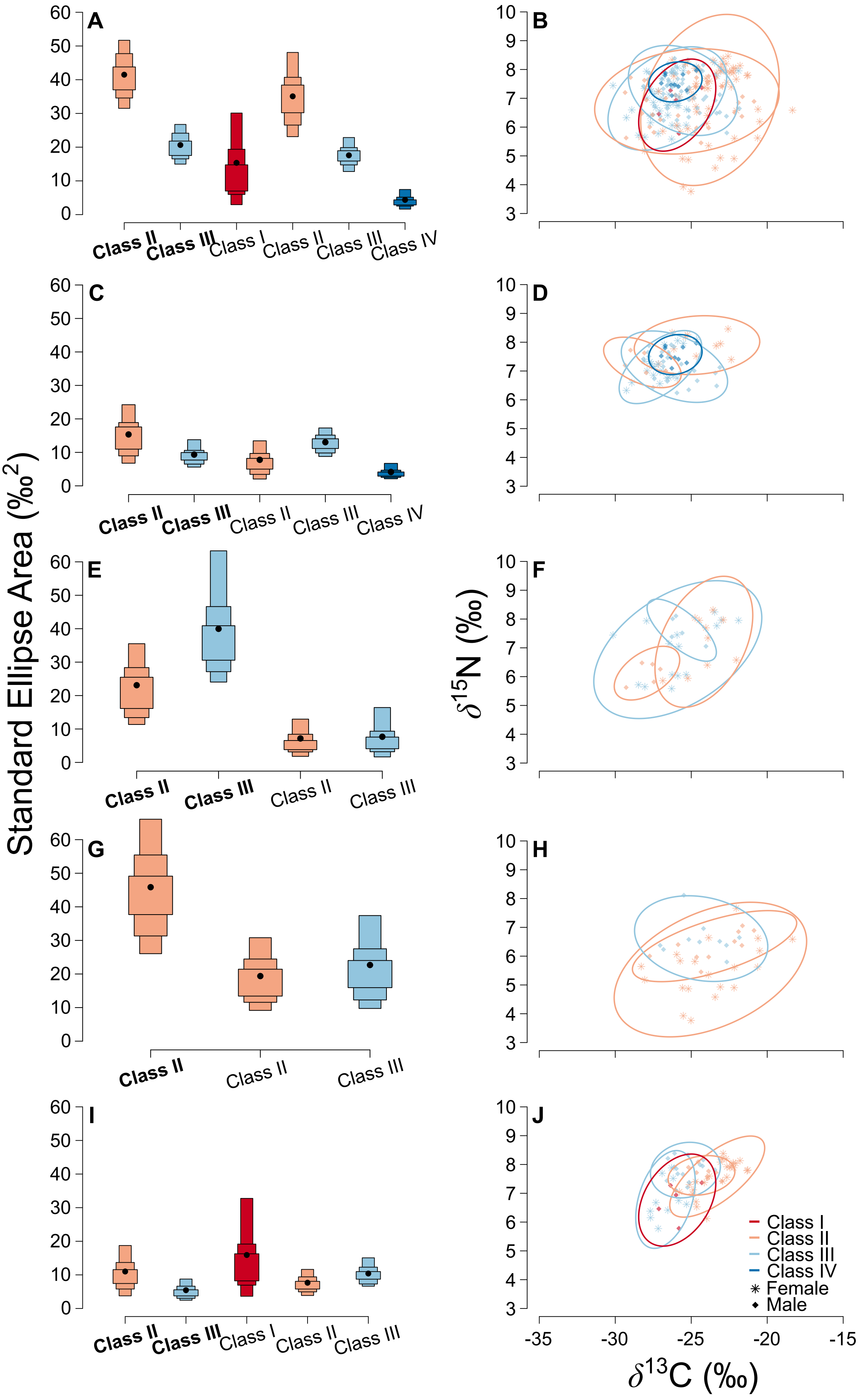

Standard Ellipse Area (‰²)
δ15N (‰)
δ13C (‰)
A
B
C
D
E
F
G
H
I
J
Class II
Class III
Class I
Class IV
Female
Male
